# Random effects structure for testing interactions in linear mixed-effects models

**DOI:** 10.3389/fpsyg.2013.00328

**Published:** 2013-06-05

**Authors:** Dale J. Barr

**Affiliations:** Institute of Neuroscience and Psychology, University of GlasgowGlasgow, UK

In a recent paper on mixed-effects models for confirmatory analysis, Barr et al. (2013) offered the following guideline for testing interactions: “one should have by-unit [subject or item] random slopes for any interactions where all factors comprising the interaction are within-unit; if any one factor involved in the interaction is between-unit, then the random slope associated with that interaction cannot be estimated, and is not needed” (p. 275). Although this guideline is technically correct, it is inadequate for many situations, including mixed factorial designs. The following new guideline is therefore proposed: *models testing interactions in designs with replications should include random slopes for the highest-order combination of within-unit factors subsumed by each interaction.* Designs with replications are designs where there are multiple observations per sampling unit per cell. Psychological experiments typically involve replicated observations, because multiple stimulus items are usually presented to the same subjects within a single condition. If observations are not replicated (i.e., there is only a single observation per unit per cell), random slope variance cannot be distinguished from random error variance and thus random slopes need not be included.

This new guideline implies that a model testing *AB* in a 2 × 2 design where *A* is between and *B* within should include a random slope for *B*. Likewise, a model testing all two- and three- way interactions in a 2 × 2 × 2 design where *A* is between and *B*, *C* are within should include random slopes for *B*, *C*, and *BC*.

The justification for the guideline comes from the logic of mixed-model ANOVA. In an ANOVA analysis of the 2 × 2 design described above, the appropriate error term for the test of *AB* is *MS*_*UB*_, the mean squares for the unit-by-*B* interaction (e.g., the subjects-by-*B* or items-by-*B* interaction). For the 2 × 2 × 2 design, the appropriate error term for *ABC* and *BC* is *MS*_*UBC*_, the unit-by-*BC* interaction; for *AB*, it is *MS*_*UB*_; and for *AC*, it is *MS*_*UC*_.

To what extent is this ANOVA logic applicable to tests of interactions in mixed-effects models? To address this question, Monte Carlo simulations were performed using R (R Core Team, 2013). Models were estimated using the lmer() function of lme4 (Bates et al., 2013), with *p*-values derived from model comparison (α = 0.05). The performance of mixed-effects models (in terms of Type I error and power) was assessed over two sets of simulations, one for each of two different mixed factorial designs. The first set focused on the test of the *AB* interaction in a 2 × 2 design with *A* between and *B* within; the second focused on the test of the *ABC* interaction in a 2 × 2 × 2 design with *A* between and *B*, *C* within. For simplicity all datasets included only a single source of random effect variance (e.g., by-subject but not by-item variance). The number of replications per cell was 4, 8, or 16. Predictors were coded using deviation (−0.5, 0.5) coding; identical results were obtained using treatment coding. In the rare case (~2%) that a model did not converge, it was removed from the analysis. Power was reported with and without adjustment for Type I error rate, using the adjustment method reported in Barr et al. (2013).

For each set of simulations at each of the three replication levels, 10,000 datasets were randomly generated, each with 24 sampled units (e.g., subjects). The dependent variable was continuous and normally distributed, with all data-generating parameters drawn from uniform distributions. Fixed effects were either between −2 and −1 or between 1 and 2 (with equal probability). The error variance was fixed at 6, and the random effects variance/covariance matrix had variances ranging from 0 to 3 and covariances corresponding to correlations ranging from −0.9 to 0.9.

For the 2 × 2 design, mixed-effects models with two different random effects structures were fit to the data: (1) by-unit random intercept but no random slope for *B* (“RI”), and (2) a maximal model including a slope for *B* in addition to the random intercept (“Max”). For comparison purposes, a test of the interaction using mixed-model ANOVA (“AOV”) was performed using R's aov() function.

Results for the test of the *AB* interaction in the 2 × 2 design are in Tables 1 and 2. As expected, the Type I error rate for ANOVA and maximal models were very close to the stated α-level of 0.05. In contrast, models lacking the random slope for *B* (“RI”) showed unacceptably high Type I error rates, increasing with the number of replications. Adjusted power was comparable for all three types of analyses (Table 2), albeit with a slight overall advantage for RI.

**Table 1:** **Type I error rate for the test of *AB* in the 2 × 2 design**.

**Reps**	**RI**	**Max**	**AOV**
4	0.170	0.063	0.050
8	0.267	0.064	0.052
16	0.395	0.063	0.049

**Table 2:** **Power for the test of *AB* in the 2 × 2 design, Adjusted (Raw) *p*-values**.

**Reps**	**RI**	**Max**	**AOV**
4	0.495 (0.704)	0.469 (0.507)	0.471 (0.471)
8	0.594 (0.847)	0.558 (0.604)	0.558 (0.565)
16	0.649 (0.922)	0.619 (0.657)	0.619 (0.619)

The test of the *ABC* interaction in the 2 × 2 design was evaluated under four different random effects structures, all including a random intercept but varying in which random slopes were included. The models were: (1) random intercept only (“RI”); (2) slopes for *B* and *C* but not for *BC* (“nBC”); (3) slope for *BC* but not for *B* nor *C* (“BC”); and (4) maximal (slopes for *B*, *C*, and *BC*; “Max”).

For the test of the *ABC* interaction, ANOVA and maximal models both yielded acceptable Type I performance (Table 3); the model with the *BC* slope alone (“BC”) was comparably good. However, the model excluding the *BC* slope had unacceptably high Type I error rates; surprisingly, omitting this random slope may be even worse than a random-intercept-only model. Adjusted power was comparable across all analyses (Table 4).

**Table 3:** **Type I error rate for test of *ABC* in 2 × 2 × 2 design**.

**Reps**	**RI**	**nBC**	**BC**	**Max**	**AOV**
4	0.069	0.102	0.050	0.046	0.046
8	0.124	0.159	0.059	0.057	0.051
16	0.197	0.241	0.063	0.062	0.052

**Table 4:** **Power for test of *ABC* in 2 × 2 × 2 design, Adjusted (Raw) *p*-values**.

**Reps**	**RI**	**nBC**	**BC**	**Max**	**AOV**
4	0.422 (0.478)	0.418 (0.546)	0.396 (0.397)	0.412 (0.412)	0.405 (0.405)
8	0.562 (0.711)	0.567 (0.753)	0.552 (0.575)	0.564 (0.578)	0.554 (0.557)
16	0.649 (0.866)	0.651 (0.889)	0.653 (0.690)	0.657 (0.687)	0.656 (0.661)

To summarize: when testing interactions in mixed designs with replications, it is critical to include the random slope corresponding to the highest-order combination of within-subject factors subsumed by each interaction of interest. It is just as important to attend to this guideline when one seeks to simplify a non-converging model as when one is deciding on what structure to fit in the first place. Failing to include the critical slope in the test of an interaction can yield unacceptably high Type I error rates. Indeed, a model that includes all relevant random slopes except for the single critical slope may perform just as badly as (or possibly even worse than) a random-intercepts-only model, even though such a model is nearly maximal. Finally, note that including only the critical random slope in the model was sufficient to obtain acceptable performance, as illustrated by the “BC” model in the 2 × 2 × 2 design.

Although the current simulations only considered interactions between categorical variables, the guideline applies whenever there are replicated observations, regardless of what types of variables are involved in an interaction (e.g., continuous only, or a mix of categorical and continuous). For example, consider a design with two independent groups of subjects, where there are observations at multiple time points for each subject. When testing the time-by-group interaction, the model should include a random slope for the continuous variable of time; if time is modeled using multiple terms of a polynomial, then there should be a slope for each of the terms in the polynomial that interact with group. For instance, if the effect of time is modeled as *Y* = β_0_ + β_1_ *t* + β_2_ *t*^2^ and the interest is in whether the β_0_ and β_1_ parameters vary across group, then the random effects structure should include slopes for both the group-by-*t* and group-by-*t*^2^ interactions.
